# Introduction to the *RSC Advances* themed collection *Chemistry in Biorefineries*

**DOI:** 10.1039/d3ra90087h

**Published:** 2023-09-29

**Authors:** Carlos Martín, Alejandro Rodríguez, Fabio Montagnaro

**Affiliations:** a Department of Biotechnology, Inland Norway University of Applied Sciences N-2317 Hamar Norway carlos.medina@inn.no; b Department of Chemistry, Umeå University SE-901 87 Umeå Sweden; c BioPrEn Group, Instituto Químico para la Energía y el Medioambiente, Chemical Engineering Department, Universidad de Córdoba 14014 Córdoba Spain a.rodriguez@uco.es; d Department of Chemical Sciences, University of Naples Federico II, Complesso Universitario di Monte Sant’Angelo 80126 Naples Italy fabio.montagnaro@unina.it

## Abstract

Professor Carlos Martín, Professor Alejandro Rodríguez and Professor Fabio Montagnaro introduce the *RSC Advances* themed collection *Chemistry in Biorefineries*.
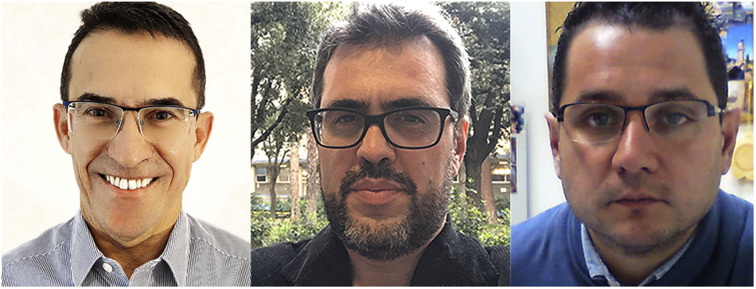

## Introduction

The biorefinery concept, which takes traditional refineries as a starting point and adapts them to environmentally friendly processes based on bioresources as raw materials, has attracted significant scientific interest during the last few years.^1^ Biorefineries are a realistic alternative for producing the advanced biofuels, bio-based materials, and chemicals required in a post-petroleum scenario.^2^ The residual lignocellulosic biomass generated by agriculture, forestry, crop processing, and other industries is a major feedstock base for biorefineries. Biomass abundance, low cost, and composition provide the required raw materials for the sustainable development of society without depending on fossil-based resources.^3^ Setting up efficient biorefineries requires a deep understanding of the chemistry behind the biorefining processes. This themed collection aims to deepen the current knowledge of chemistry in biorefineries.

## This collection

The themed collection *Chemistry in biorefineries* features 15 contributions, including 13 original research papers and 2 review articles. The contributions come from almost all the main geographical areas of the world, from North to South America, from Europe to Africa and Asia, and cover recent developments in the chemistry of lignocellulose components, methods for fractionation of lignocellulosic biomass, and chemical conversion processes occurring in biorefineries.

Biomass fractionation is a core step for valorisation of the main constituents following a complete biorefinery approach.^4^ In this collection, three articles contribute to enhancing the understanding of biomass fractionation. Monção *et al.* report the fractionation of fibres of the halophyte plant *Salicornia dolichostachya* by organosolv pretreatment (https://doi.org/10.1039/D2RA04432C). By carefully controlling the process parameters, cellulose-rich pretreated solids were produced, and high removal of hemicelluloses and lignin were achieved. The obtained cellulose was completely hydrolysable, the lignin fraction had high purity, and the hemicelluloses were recovered as a separate product consisting mostly of oligosaccharides. Ovejero-Pérez *et al.* introduced an autohydrolysis step prior to ionosolv treatment of *Eucalyptus globulus* biomass, which resulted in an efficient separation of hemicelluloses, cellulose, and lignin (https://doi.org/10.1039/D2RA08013C). Lemma *et al.* effectively separated hemicellulosic sugars and cellulose-rich fibre from enset (*Ensete ventricosum*) biomass by combining steaming pretreatment with soda pulping (https://doi.org/10.1039/D2RA07220C).

Hydrothermal pretreatment is an effective method for lignocellulose fractionation,^5^ but it cannot avoid the formation of inhibitors of the enzymatic saccharification and the microbial fermentation, which are two major operations in biorefining.^6^ In this collection, Wu *et al.* reported the conditioning of a birch pretreatment liquor by liquid–liquid extraction using long-chain organic extractants (LCOE) for improving fermentation and saccharification (https://doi.org/10.1039/D3RA02210B). The study compared the effectiveness of three LCOE to that of two conventional organic solvents. The investigation showed that the conditioning with LCOE, which can be performed at room temperature and acidic pH, promotes both the fermentability of hydrolysates and the enzymatic saccharification of cellulose.

In a biorefinery environment, lignin, which is the major aromatic constituent of lignocellulosic biomass, can be valorised to aromatics, polymers, biofuels, and biomaterials.^7,8^ This collection features four contributions dealing with lignin characterisation and valorisation. Li *et al.* present an assessment of the opportunities and challenges of catechyl lignin (C-lignin), a recently discovered biopolymer, whose homogeneous linear structure facilitates chemical conversion and provides new valorisation perspectives (https://doi.org/10.1039/D3RA01546G). The review summarises the biosynthesis of C-lignin in plants, provides an overview on its isolation and on various depolymerisation approaches, and explores new application areas based on its unique structure. The advantages and drawbacks of different methods for C-lignin isolation are discussed, the potential of treatments with deep eutectic solvents is highlighted, and the reductive catalytic fractionation as an emerging technology for effective depolymerisation is addressed.

Lignin’s versatile chemistry allows many reactions through its multiple functional groups.^9^ That enables addition of different functionalities, which provide specific properties that make it suitable for replacing fossil-based polymers. In a review on lignin applications, Ruwoldt *et al.* summarise the state of the art on the development of lignin-based functional surfaces, films, and coatings, with a focus on the formulation and final uses (https://doi.org/10.1039/D2RA08179B). The article discusses the potential of technical lignins, a currently under utilised by-product of pulping and biorefinery processes, which has a huge potential for chemical modification and upgrading to different material applications.

Although poplar is a hardwood tree of major importance for biorefinery applications,^10^ the structure of lignin from different organs has so far not been investigated to a comparable extent. Stem lignin has been well studied, while foliar lignin has been less well studied. In this collection, original research by Bryant *et al.* discloses new knowledge on the chemistry of poplar foliar lignin (https://doi.org/10.1039/D3RA03142J). A set of 11 *Populus trichocarpa* foliage samples was characterised using advanced analytical techniques. Clear differences between foliage and stem tissues were revealed using advanced analytical techniques. Heteronuclear single-quantum coherence nuclear magnetic resonance and Fourier-transform infrared spectroscopy revealed high variability in lignin structure, while gas chromatography-mass spectrometry showed a high degree of metabolite abundance.

Another lignin-related contribution of interest is a solid-state chemical modification protocol without external gas supply nor liquid reactants, developed by Wurzer *et al.* to generate N-lignins (https://doi.org/10.1039/D3RA00691C). The new protocol allows the performance of the lignin modification in closed continuous reactor systems, and it is expected to widen the possibilities of using N-lignins as an organic fertiliser or soil amendment.

Spent coffee ground (SCG) is an agro-industrial waste with potential as feedstock for biorefineries.^11^ The controlled hydrolysis of galactoglucomannan, the main SCG carbohydrate, leads to the formation of mannooligosaccharides (MOS), which have health-promoting effects due to their prebiotic and antioxidant activity.^12^ Magengelele *et al.* reported MOS production from SCGs by alkaline pretreatment followed by hydrolysis with a *Bacillus* sp. derived *endo*-β-1,4-mannanase (https://doi.org/10.1039/D2RA07605E). An *in vitro* evaluation of the product showed its prebiotic effect on beneficial bacteria, *e.g.*, *Lactobacillus bulgaricus*, *Bacillus subtilis* and *Streptococcus thermophilus*. Assays performed under simulated gastric conditions revealed that the product is suitable for oral administration.

The Guest Editors would like to thank all the experts that have authored the articles included in this themed collection. Their high-quality contributions provide highly valuable new insights that will be well appreciated by readers interested in the fascinating area of the chemistry of biorefining processes.

## Author contributions

Conceptualization and methodology: C. M., A. R., F. M.; writing—original draft preparation, review and editing: C. M., A. R., F. M.

## Conflicts of interest

There are no conflicts to declare.

## Supplementary Material
